# A small-angle scattering model for broad correlation peaks and small diffuse scattering

**DOI:** 10.1107/S1600576726006825

**Published:** 2026-07-31

**Authors:** Henrich Frielinghaus

**Affiliations:** a Jülich Centre for Neutron Science JCNS at MLZ, Forschungszentrum Jülich GmbH, 85748 Garching, Germany; Argonne National Laboratory, USA

**Keywords:** porous structures, hierarchical structures, ultra-small-angle neutron scattering, small-angle neutron scattering, ultra-small-angle X-ray scattering, small-angle X-ray scattering, small-angle light scattering

## Abstract

Porous and/or bicontinuous structures often display a pronounced correlation peak in their small-angle scattering that indicates a preferred domain spacing *d*. A first attempt to describe the full *q* dependence of small-angle scattering profiles has been formulated in a previous publication [Frielinghaus (2026) *J. Appl. Cryst.***59**, 837–844]. However, the diffuse scattering, *i.e.* the forward scattering, is often found to be lower than in this simple framework, while the correlation peak is rather broad. By adding one more parameter σ that stretches the correlation peak with respect to the residual features of the theory, a more general heuristic theoretical framework for porous and/or bicontinuous structures is obtained. The predominant conditions and their physical meaning are discussed in context with practical examples.

## Abstract 2

For porous and/or bicontinous structures with rather broad correlation peaks and small diffuse scattering, a heuristic theory makes the connection to conditions of material production.

## 1. Introduction

In a recent publication (Frielinghaus, 2026*b* ▸), I generalized the microemulsion-like scattering theory for small-angle scattering (Hamley, 2021 ▸; Roe, 2000 ▸; Gommes *et al.*, 2021 ▸), *i.e.* the classical Teubner–Strey theory (Teubner & Strey, 1987 ▸), to arbitrary dimensions *n*. While there was a pronounced scattering peak described by the structural parameters of the domain spacing *d* (with a preferential wavevector of magnitude *k*_0_ = 2π/*d*) and the correlation length ξ, a Porod scattering is also found at the high-*q* end according to *q*^−1−*n*^. In this framework, the diffuse scattering, *i.e.* the forward scattering, is tightly connected to the two structural parameters. This means that the different levels of the diffuse and peak scattering are not completely independent. In the case of microemulsions, there is absolutely no need to question this dependence because the Teubner–Strey theory arises from a functional series of the thermodynamic potential (a Landau theory) that connects to the scattering function through the fluctuation-dissipation theory. But these arguments from statistical physics do not necessarily need to hold for arbitrary porous materials that underwent a chemical process that may have been far from thermal equilibrium. Often, pronounced broad correlation peaks are observed, while the diffuse scattering is low compared with the generalized Teubner–Strey formalism. This scenario is the issue of the current article.

A rather general approach (Levitz & Tchoubar, 1992 ▸) is achieved by describing the two independent statistics of chords (*i.e.* their length distribution) in the solid and pore phases (or in two domains). At the general level, the statistics can be obtained from real-space micrographs and then be compared with complementary scattering profiles. So the two statistical functions are not necessarily parameterized and a huge variety of scattering functions can be obtained. However, the reduction to a few parameters from experimental scattering curves is desirable, and then different experiments and materials can be compared on the same level. The original publication (Levitz & Tchoubar, 1992 ▸) introduced a parameterization that resulted in the Debye–Büche formula (Koberstein & Stein, 1980 ▸). Other parameterizations that describe a correlation peak have not been discussed so far.

A rather sophisticated advantage of the Teubner–Strey generalization (Frielinghaus, 2026*b* ▸) is that one obtains the bulk with the corresponding surface scattering. That was motivated by the appearance of coherent multiple scattering (Frielinghaus & Gommes, 2025 ▸), a specific situation for quite small *q* [in terms of ultra-small-angle neutron scattering (USANS) and ultra-small-angle X-ray scattering (USAXS) (Barker *et al.*, 2005 ▸; Magerl *et al.*, 2024 ▸; Ji *et al.*, 2022 ▸; Zhang & Ilavsky, 2010 ▸)] and relatively high contrasts [but which may also be found for small-angle light scattering (SALS) experiments (Nishida *et al.*, 2008 ▸)], where surface scattering replaces the classical bulk scattering. The case of coherent multiple scattering has to be seen in context with the anomalous surface reflection described by Yoneda and Vineyard (Yoneda, 1963 ▸; Guentert, 1965 ▸; Vineyard, 1982 ▸) where resonances at surfaces at shallow incident angles highlight the surfaces as such. All of this is discussed in full length in the original publication.

In this paper, I extend the current set of parameters, *i.e.* the domain spacing *d*, the correlation length ξ, the dimensionality *n*, and a scaling factor or amplitude *A*_2_, by adding a fifth parameter, a stretching factor σ. This parameter allows for stretching of the correlation peak with respect to the diffuse scattering level.

First, the current state of the generalized Teubner–Strey theory (Frielinghaus, 2026*b* ▸) is briefly summarized. Here, the asymptotic scattering function for small and large *q*, *i.e.* for the diffuse scattering and the Porod scattering, could be separated from the peak function. Secondly, the stretching of the peak function is introduced. In this context, the connection to the real-space correlation function is discussed and the conditions for broad peaks with small diffuse scattering are elaborated. Then, a few scattering curves from the literature are modelled and the resulting parameters are discussed. A summary at the end wraps up the most important findings of this article.

## 2. The current state

For porous and bicontinuous materials in arbitrary (fractional) dimensions *n*, I have derived scattering functions for bulk and surface scattering (Frielinghaus, 2026*b* ▸). When normalizing the scattering function to unity in the limit of small scattering vector magnitudes *q*, I arrive at
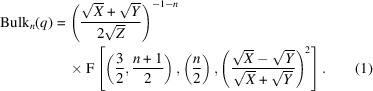
Here, the following abbreviations are defined, namely *X* = (*k*_0_ + *q*)^2^ + ξ^−2^, *Y* = (*k*_0_ − *q*)^2^ + ξ^−2^ and *Z* = 
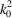
 + 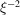. The structural sizes are given in terms of the preferential wavevector *k*_0_ = 2π/*d*, with the domain spacing *d* and the correlation length ξ. The first term in brackets in equation (1) is connected to the asymptotic scattering at small and large *q*. It levels off at unity for small scattering angles and therefore represents a certain level of diffuse scattering. The asymptote at high scattering angles *q* is connected to a power law *q*^−1−*n*^ that describes the Porod scattering in *n* dimensions. The second factor is given by the ordinary Gauss hypergeometric function, 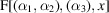 ≡ 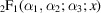. Its argument 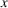
 = 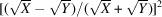 is asymptotically zero for small and large *q*, so the whole factor also takes the asymptotic value unity. For intermediate scattering vector magnitudes *q* ≃ *k*_0_, the factor describes a peak that is (in logarithmic representation) set on top of the correct asymptotic function represented by the first term. With this theory, the diffuse scattering relative to the peak intensity at *q* ≃ *k*_0_ is *not independent* of the two structural parameters *d* and ξ. For a small-angle scattering profile in absolute units, a set of factors have to be considered to describe the macroscopic scattering cross section, 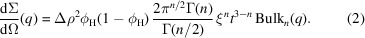
The contrast Δρ is connected to the scattering length density difference between the two materials (or between the solid material and pores in the case of a porous material). The volume fraction ϕ_H_ is connected to the hydrogenous material in the case of neutron scattering but also refers to either material for other probes. Here, the correlation volume ξ^*n*^*t*^3−*n*^ comes into play, with *t* being the thickness of the building blocks. The latter factor represents the contribution to the volume where no correlation is found. These building blocks make up the fractal structure that is described by the high-*q* end of Bulk_*n*_(*q*), and in principle a respective Guinier factor referring to *t* would need to be introduced here to describe a high-*q* cutoff.

For simplicity, the connections to the simplest case of *n* = 3 dimensions will be discussed here, a frequent case for which often only small corrections need to be considered. At a later stage of the paper, the corrections will be discussed with resepect to the dominating terms. However, the hyper­geometric function in the case *n* = 3 reads F[(3/2, 2), (3/2), *x*] = (1 − *x*)^−2^. Then equation (1) can be simplified to
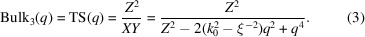
Of course, this results in the well known Teubner–Strey expression (Teubner & Strey, 1987 ▸), which does not need a deeper discussion. Therefore, I refer to this expression as TS(*q*).

As a second function, the underlying asymptotic function is defined as
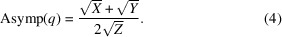
In the limit of an infinitely large correlation length ξ, this function is linear with |*q*/*k*_0_| for |*q*| > *k*_0_ and unity in the central part with |*q*| < *k*_0_. The correlation length ξ smooths the sharp edges and, therefore, contributes to a more physical and realistic appearance. In the original expression of equation (1), the respective power Asymp^−1−*n*^(*q*) then describes the correct Porod scattering.

For the corresponding surface scattering, a similar expression with a hypergeometric function was derived: 
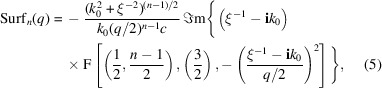
with a small correction by the factor 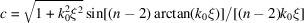
close to unity. The whole expression contains a complex argument with **i** being the imaginary unit. Again, the expression is normalized to unity for small scattering angles *q*. For the calibrated scattering cross section, one obtains the following:
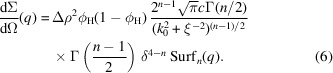
Together, equation (5) and (6) describe the film scattering of microemulsions in *n* = 3 dimensions. The contrast and the volume fraction ϕ_H_ refer now to the film material (surfactant in the case of microemulsions). Here, the thickness δ describes the surfactant film thickness, but more generally δ describes the thickness of a building block perpendicular to the fractal that is described by the high-*q* end of Surf_*n*_(*q*). Again, as for the bulk scattering, in principle another Guinier factor would be needed here. The factor 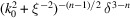 with the plain numerical factors of equation (6) is the respective correlation patch size of this description. For microemulsions, an additional term for the osmotic compressibility, *A*_compr_/(1 + *q*^2^ξ^2^/4) was added (Frielinghaus & Gommes, 2025 ▸). In the following, this term will be neglected because the emphasis here is on porous materials. In the exact case of *n* = 3, equation (5) reduces to (Frielinghaus & Gommes, 2025 ▸; Frielinghaus, 2026*b* ▸) 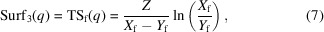
with the abbreviations *X*_f_ = (*k*_0_ + *q*/2)^2^ + ξ^−2^ and *Y*_f_ = (*k*_0_ − *q*/2)^2^ + ξ^−2^. The index ‘f’ indicates the film or surface scattering in all these expressions. Similarly to above, an asymptotic function can be defined:
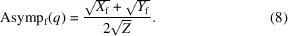
For film scattering, it is not directly evident from equation (5) that this is the correct asymptotic function. So at the current level, I keep this as a heuristic definition.

**Figure 1:**
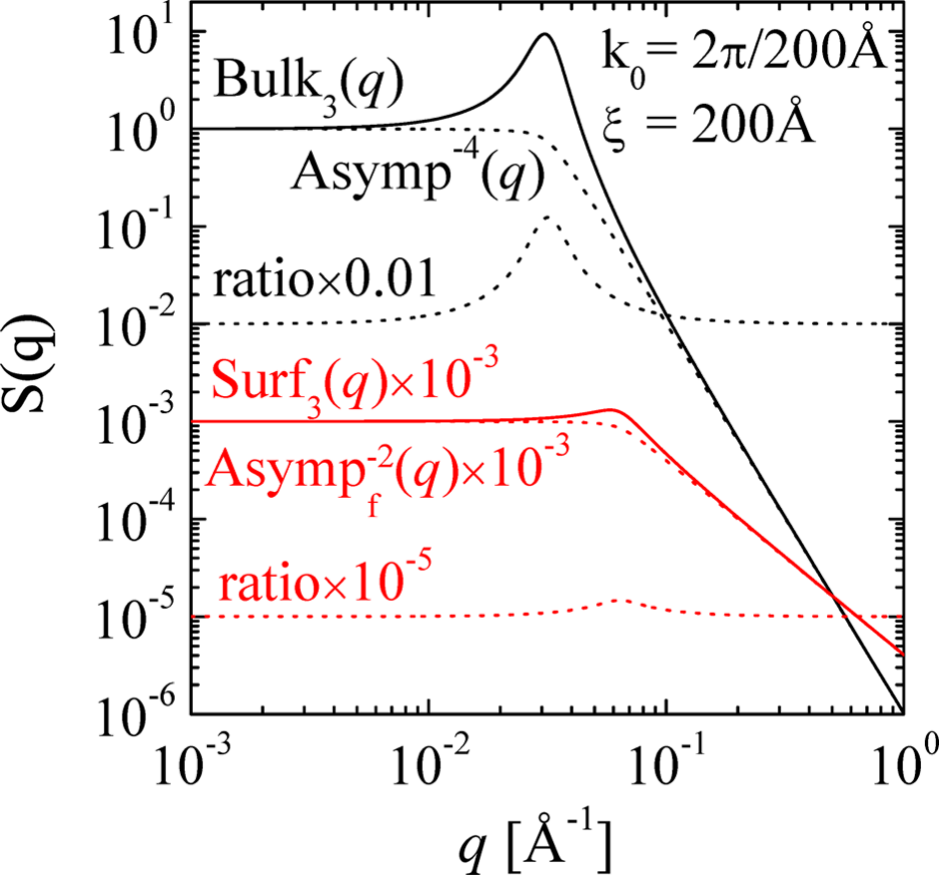
The different contributions to the scattering functions Bulk_3_(*q*) (solid black line) and Surf_3_(*q*) (solid red line) for *n* = 3 dimensions (double logarithmic scale). The asymptotes Asymp^−4^(*q*) (dashed black line) and 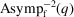 (dashed red line) are on the same scale. The isolated peak functions are obtained from the ratios (also dashed black and red lines, but shifted down). In the case of bulk scattering this ratio is obtained from the hypergeometric function in equation (1). In contrast, the surface scattering does not find an easily separable factor in the current formulation.

**Figure 2:**
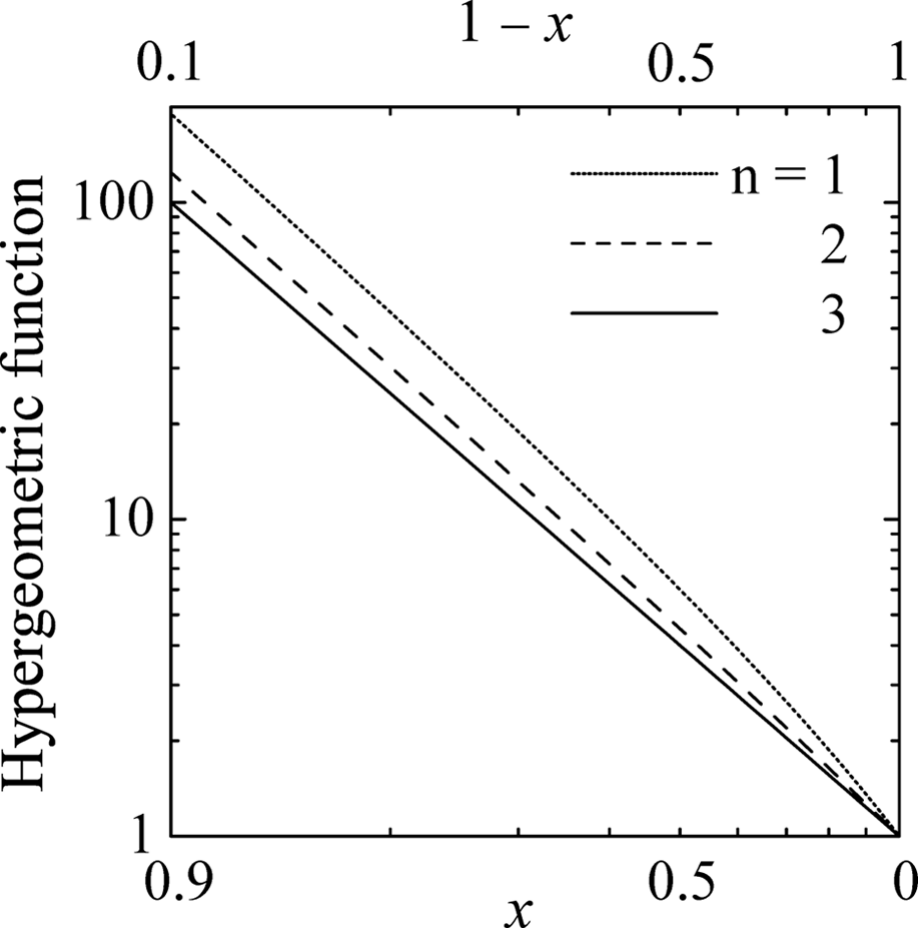
The hypergeometric function F{[3/2, (*n* + 1)/2], (*n*/2), *x*} as a function of *x* in a double logarithmic representation for different dimensionalities *n*. Note that the *x* axis is correctly scaled for 1 − *x* (upper scale), in contrast to the simple scale of *x* (lower scale). Asymptotically, for all *n* the functions scale with (1 − *x*)^−2^ for *x* → 1.

The different contributions of the asymptotes and peak functions to the overall scattering functions Bulk_3_(*q*) = TS(*q*) and Surf_3_(*q*) = TS_f_(*q*) are depicted in Fig. 1 ▸. One can see the asymptotes for small and large *q* at the same scale. In the peak region *q* ≃ *k*_0_, the asymptotes bend smoothly between the two linear regions (on this double logarithmic scale). The ratios then represent the pure peak functions. In the case of bulk scattering, the peak function is represented by the hyper­geometric function in equation (1). Already from the argument 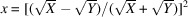 a peaked function is obtained. The hypergeometric function only translates this scale of *x* to the physically motivated scattering function in terms of scattering intensity. A plot of this translation is given in Fig. 2 ▸ for different dimensionalities *n*. One can see here that, close to *n* = 3, the dependence on *n* is only weak and the ideal dependence (1 − *x*)^−2^ dominates. For surface scattering, a comparable separation is not intuitively seen from the appearance of equation (5). Possibly, more elaborate expressions might find a natural factorization in the future. The peak functions level off at unity for small and large *q*.

## 3. Stretching

So far, the current theory is highly motivated by the real-space correlation functions that describe an oscillatory factor for alternating domains and an exponential decay for the loss of correlations at large distances. At this level, the motivation is strictly physical and involves only two parameters, namely the domain spacing *d* and the correlation length ξ. The dis­advantage of this description is that the diffuse scattering level for *q* → 0 relative to the peak intensity is strictly related to those two structural parameters. For microemulsions, this level of understanding is sufficient and it is all motivated by statistical physics. However, for porous materials, this motivation is more heuristic and is often violated in the sense of a relatively broad peak (relatively small ξ compared with *d*) that is quite high compared with the diffuse scattering from the original expression. For that case, a stretching of the isolated peak function with a factor σ is a heuristic solution, while the asymptotes may be the same as from the original theory. A mathematical expression results in the following: 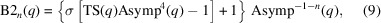

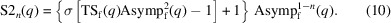
The terms in square brackets in these equations describe a simple calculation of the pure peak function for a fixed *n* = 3. These expressions use only standard functions that are available on any computer system or program without extended libraries. This approximation is in any case only intended for intermediate *x* (or ξ < *d*) and *n* ≃ 3, where I intend to describe a broader peak with little diffuse scattering. So, it is meant as an approximation in *x* which, in the context of stretching by the factor σ, has a general meaning for arbitrary dimensionalities *n* close to 3, where the corresponding hypergeometric function of the bulk scattering is not strongly dependent on *n* (Fig. 2 ▸). A similar argument I assume to be true for the surface scattering. The plain factor of the asymptote is added at the end of the expressions, here for arbitrary *n* (again, close to 3). At this point the exact dimensionality is more important, especially for the high-*q* end.

**Figure 3:**
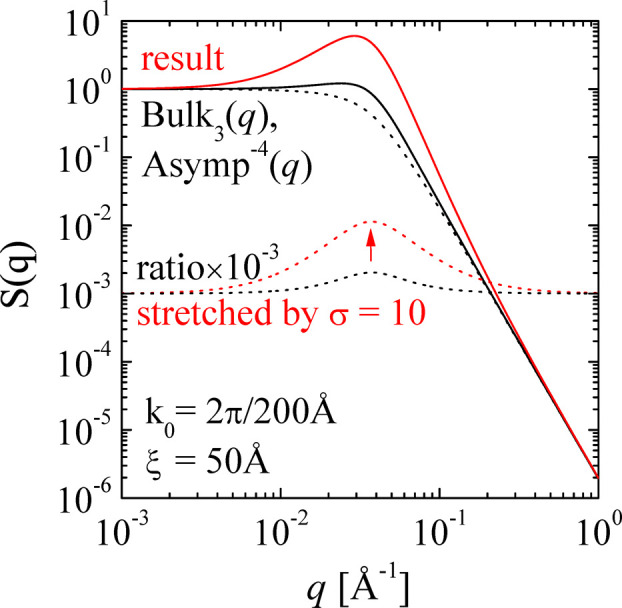
The concept of a stretched peak function is displayed here, with a relatively small correlation length ξ = 50 Å and *d* = 200 Å. The original Teubner–Strey theory is represented by the solid black line with the corresponding asymptote as a dashed line. The simple ratio is scaled down by a factor of 10^−3^ (dashed line) and a stretching factor of σ = 10 is applied (red dashed line). This stretching applied to the original scattering curve is represented by the solid red line on the original scale.

The bulk scattering function with all components with a stretching of σ = 10 is diplayed in Fig. 3 ▸. First, a standard scattering function from the original Teubner–Strey theory with a relatively small correlation length ξ = 50 Å is displayed with the corresponding asymptote on the same scale. The resulting peak function is shifted down by a factor of 10^−3^, and at that level the peak function scaled by σ = 10 is displayed in parallel (dashed black and red lines). The stretching applied to the original function is depicted by the solid red line. The peak intensity is approximately ten times stronger than the original very weak peak, while the diffuse scattering stays at the same level. This example illustrates a typical case for which the stretching is designed. At relatively small ξ, the peak is quite broad and nearly invisible on top of the asymptote. However, the stretching makes the feature of a broad peak much more dominant, and the diffuse scattering is suppressed in comparison.

**Figure 4:**
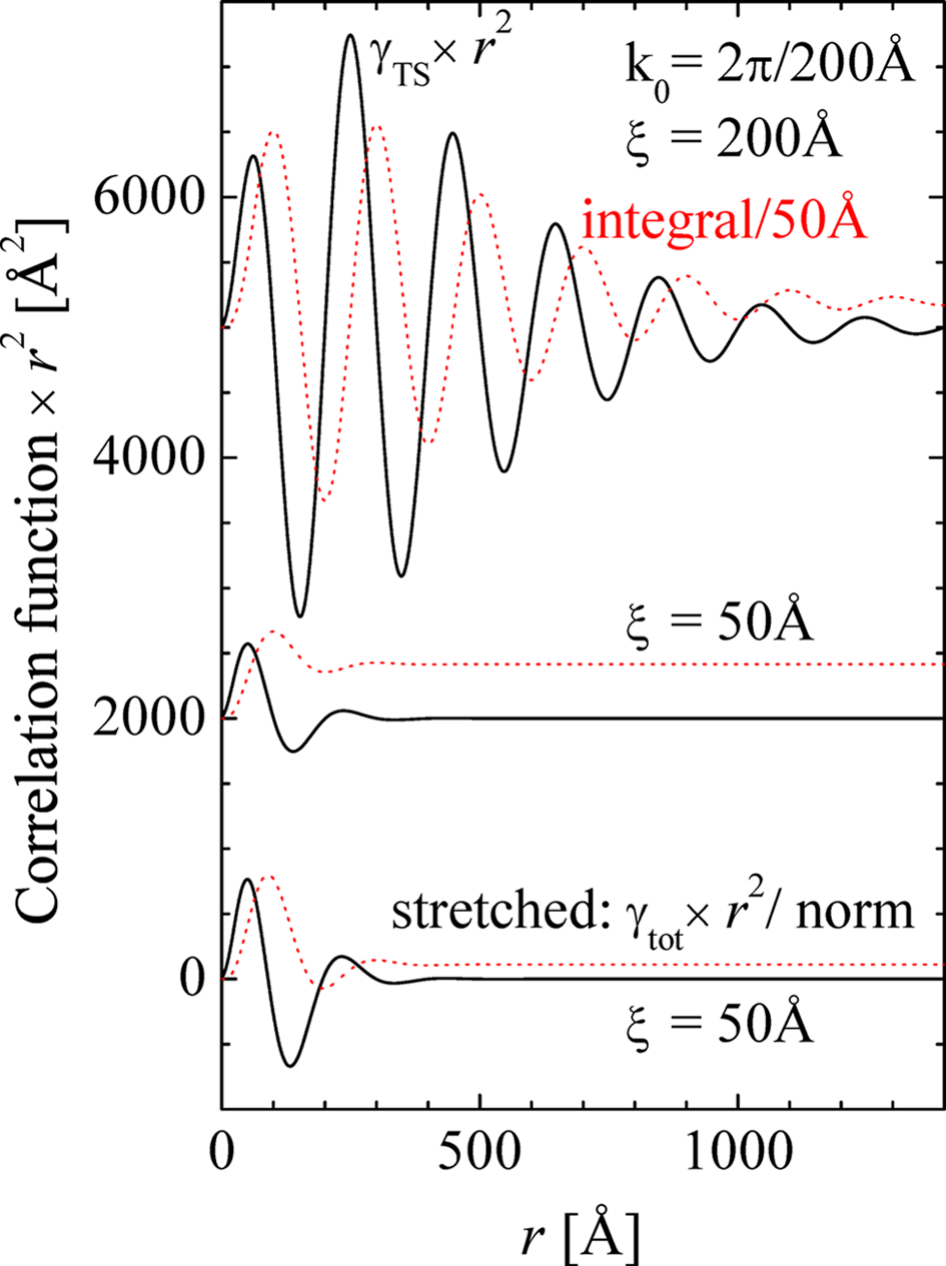
The real-space correlation functions *p*(*r*) = γ(*r*)*r*^2^ for a few example parameters (solid black lines, shifted vertically). The dashed red lines indicate the integral of the correlation function with respect to *r*, and the asymptotic value for *r* → ∞ is the forward scattering that is connected to the diffuse scattering. The top two examples are simple Teubner–Strey expressions (*d* = 200 Å and ξ = 200 Å or ξ = 50 Å). The bottom curve corresponds to a stretched scattering curve with σ = 10.

For the case *n* = 3, equation (9) simplifies further to the following expression: 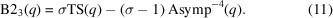
One can deduce arguments about this expression from a real-space correlation function. On this basis, the simple Teubner–Strey correlation function can be compared with the stretched function. The real-space correlation function γ(*r*) is to some extent additive. However, at the end of a calculation, a nomalization has to be considered such that γ(*r* → 0) = 1. The non-normalized correlation functions 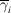 are composed according to 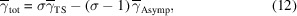
with the classical Teubner–Strey correlation function being 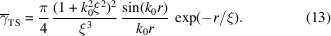
The asymptotic scattering function can be approximated by a classical Teubner–Strey scattering curve with an extremely small 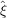 when forcing an agreement at the asymptotes *q* → 0 and *q* → ∞ and for the typical scattering vector magnitude *q* = *k*_0_. The coefficients of the polynomial denominator then read
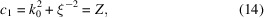

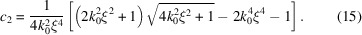
From this, one can calculate the spatial parameters of the correlation function with a very suppressed correlation length according to 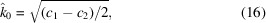

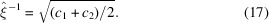
The corresponding correlation function then reads 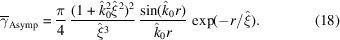
This approximation is valid for original parameters of ξ < *d*/2 (or *k*_0_ξ < π) (in the sense of being safely, *i.e.* by 30%, below). For larger correlation lengths, this approach causes artefacts in the approximation so does not apply well in such cases. One can also try to describe the asymptote for infinitely large correlation lengths (the function becomes piecewise linear): 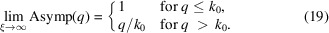
This still results in an analytical real-space correlation function, namely 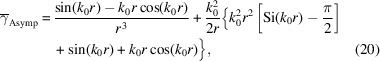
where Si(*x*) is the sine integral. However, this approach is based on an asymptote with a sharp kink that leads to stronger oscillations in the real-space correlation function for larger *r*. Going back to the first approach, a set of correlation functions are discussed in Fig. 4 ▸. The presented correlation function γ(*r*) is multiplied by the factor *r*^2^ to obtain the well known function *p*(*r*) because this weight appears when calculating the Fourier transform, *i.e.* the scattering curve. The first example with ξ = 200 Å displays many oscillations – the initial growth results from the weight *r*^2^ and the decay at larger *r* results from the exponential function of the finite correlation length. The second example with ξ = 50 Å results in only a few oscillations that immediately decay. The important example with a stretch of σ = 10 strengthens the oscillations a little bit, and the first two oscillations are highly comparable in amplitude. For the integral, this leads to a small value at large *r*, *i.e.* a small forward scattering (or diffuse scattering) compared with the pure Teubner–Strey theory (middle example). This means that the solid material correlation is just as strong as the correlation to the first neighbouring pore (or for the domain and the neighbouring domain). That is, at short distances, the structure corresponds to its inversion much more than the Teubner–Strey structure. Only a third parameter, the stretch parameter σ, can provide the desired result. One must remember that the correlation length ξ < *d*/2 is relatively small in that case. Otherwise, the diffuse scattering is suppressed in a natural way.

## 4. Practical examples

In the following a few examples of measurements with the improved modelling are discussed. The first examples look at bulk porous materials. The underlying equation for the improved modelling is given by (Frielinghaus, 2026*b* ▸)
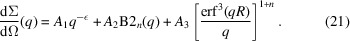
The first term is a power law for extremely large structures that fall out of the experimental window. It can be called Porod scattering of macroscopic structures. The next term is related to the improved scattering function with a stretch parameter σ [equations (9), (3) and (4) display all dependencies of B2_*n*_(*q*) on *k*_0_, ξ and σ]. This parameter often lies in the approximate range of 3 to 10. The third term relates to excess surface scattering with an identical power law to the second term [from equation (9) the exponent −1 − *n* becomes evident]. For microemulsions, it was shown that local surface roughness is not fully captured by the original Teubner–Strey theory and demands an extra term (Frank *et al.*, 2007 ▸) with the identical Porod exponent (4 in the case of microemulsions). For porous materials, a similar argument may support excess surface scattering that cannot only be related to the scale of the domains.

**Figure 5:**
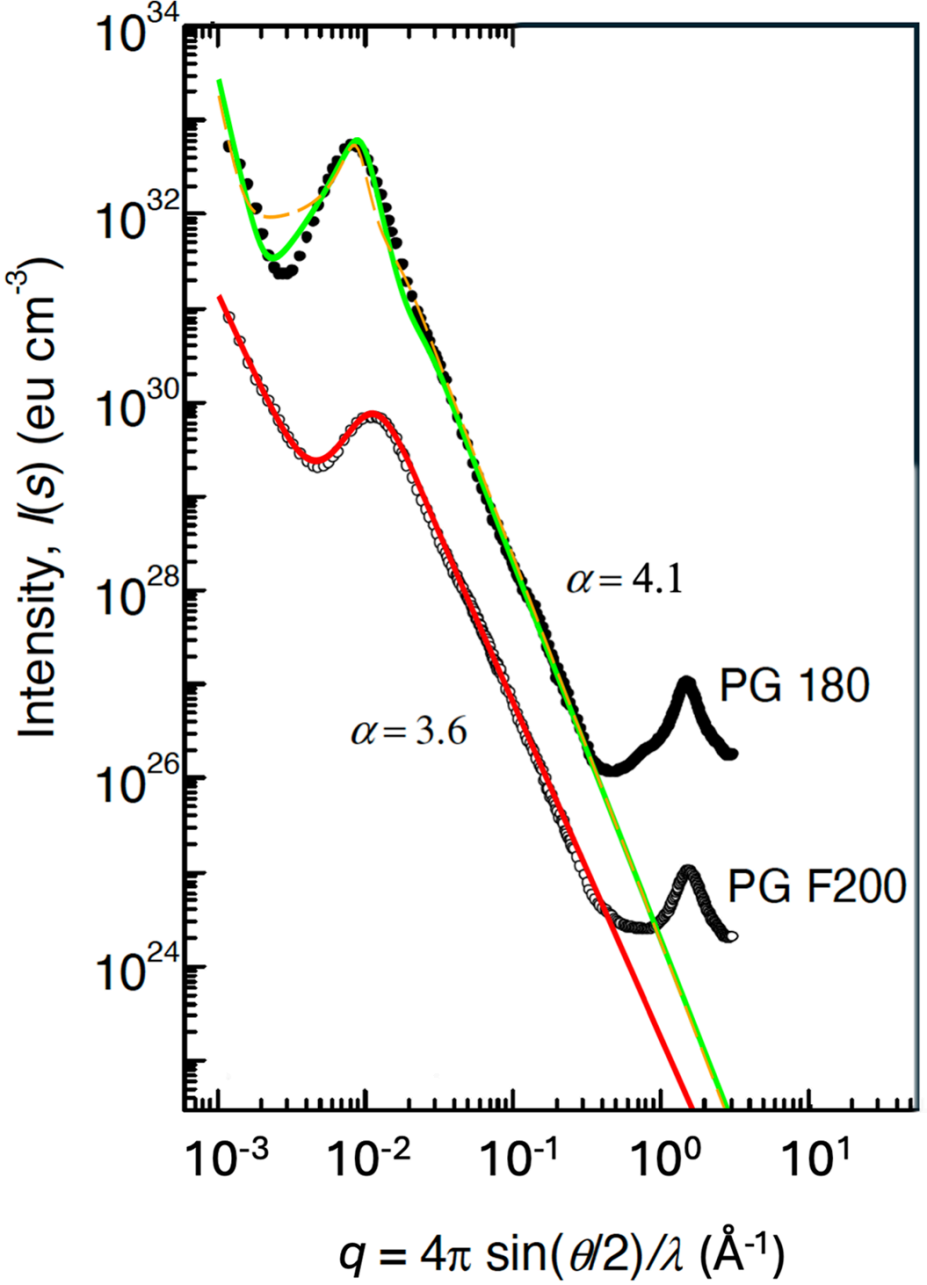
The SAXS profiles of two porous glass samples (Walter *et al.*, 2003 ▸) that were already discussed in context of the simple theory (Frielinghaus, 2026*b* ▸). The dashed orange line represents the simple modelling without the stretching parameter σ. The solid green and red lines represent the new modelling using equation (21). For the PG180 porous glass, the differences become clearly visible when modelling the first minimum around *q* = 0.003 Å^−1^. For the PGF200 glass, the simple modelling is omitted to improve clarity.

For porous glasses (Walter *et al.*, 2003 ▸), the original theory (without stretching) was already discussed (Frielinghaus, 2026*b* ▸). Now, in Fig. 5 ▸ the improvements are made visible. The figure shows the simple modelling and the modelling with a stretching parameter σ = 500 for the PG180 porous glass. One can clearly see differences when modelling the minimum around *q* = 0.003 Å^−1^. The highly suppressed forward scattering leads to a rather deep minimum that almost describes the experimental curve. For the PGF200 glass, the description is perfect and, when compared with the original fit (Frieling­haus, 2026*b* ▸), subtle improvements can also be found here. The fitting parameters are summarized in Table 1 ▸.

**Figure 6:**
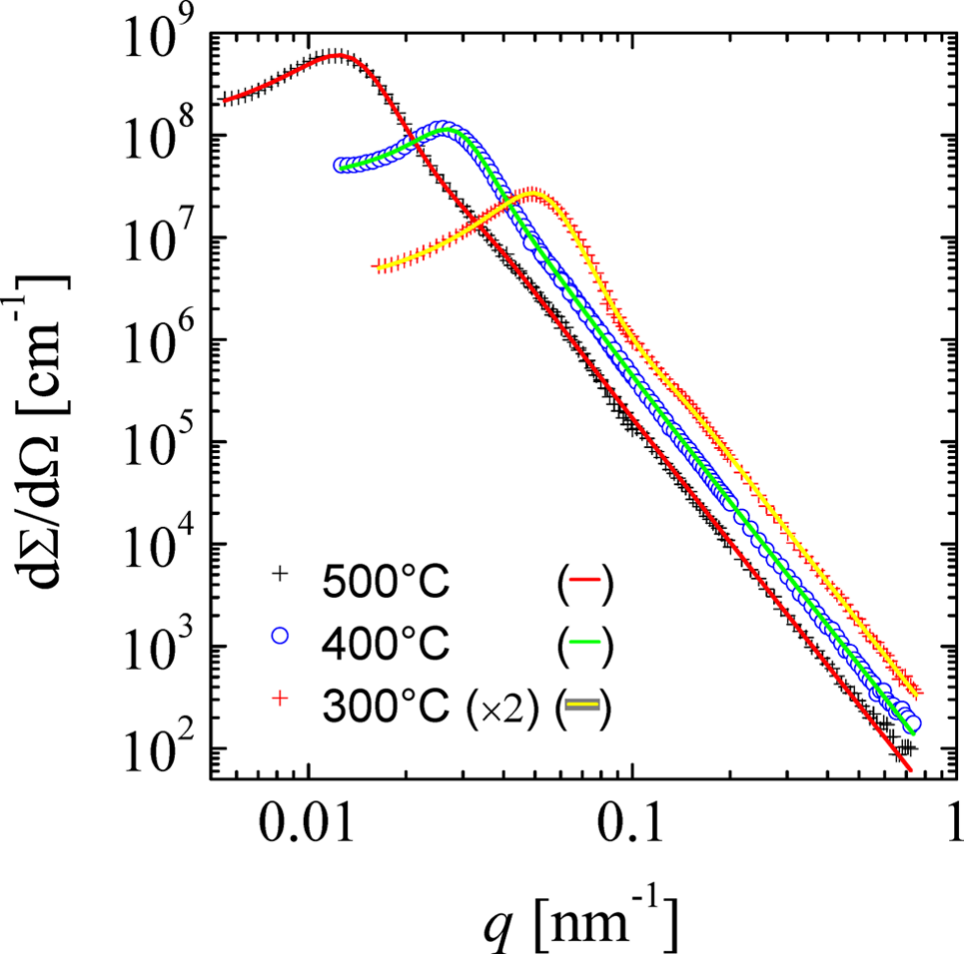
SAXS profiles for coarsened nanoporous silver–gold (NPSG) [data from Riedel *et al.* (2023 ▸)] at different annealing temperatures. The measurements are indicated by separate symbols as given in the legend. A shift factor is indicated in brackets. The modelling is depicted by continuous lines as given in the legend.

The SAXS profiles from nanoporous gold structures as described by Riedel *et al.* (2023 ▸), Shi *et al.* (2021 ▸) and Jeon *et al.* (2024 ▸) are depicted in Figs. 6 ▸ and 7 ▸. The first set (NPSG) is connected to a simple nanoporous structure with only one scale of porosity. The second set (HNPG) is connected to a hierarchical nanoporous structure with two levels of porosity. The details of the preparation and real-space micrographs can be found in the original papers. For the purpose of this article, the USAXS and SAXS curves are merged to a single data set where the USAXS data are used in preference to the SAXS data in the overlapping *q* range. One can identify a single peak for the NPSG set and two peaks for the HNPG set connected to the different scales of the hierarchy. For most of the peaks, a stretch of σ = 10 was needed to account for the relatively low diffuse scattering. Enhanced surface scattering was needed for the high-*q* tail of the curves, and only for the secondary structure in the hierarchical gold. The model description results in a very good agreement for all SAXS profiles. In the case of the first set (NPSG), the number of parameters is quite adequate. In the second case, the number of parameters is high and might be interpreted as an over-parameterization for that *q* range. However, when putting the emphasis on the structural parameters *k*_0_ (or *d*) and ξ of the different hierarchies, the model provides access to physical parameters. The strongly suppressed diffuse scattering can be interpreted as a much more regular structure than simple microemulsions.

Looking at the range of values for *k*_0_ξ with considerable stretching, the values for the present work lie between 1.8 and 3.7. This is in the range of π and below, as already obtained from the limits of equation (18), but there it was a limit for the approximation of the real-space correlation function and not a general limit. Here, the correlations of the solid and the first pore material (or one domain with the first neighbour) are strong, but they decay strongly beyond this. For much lower ξ values the peak would disappear anyway, and so a proper peak model becomes obsolete. The range for values of *k*_0_*R* lies between approximately 0.5 and 2. From that range, we can derive a practical range of values for 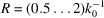 that keep the excess surface scattering reasonably low in comparison with the dominating peak scattering.

Comparing the scattering amplitude *A*_2_ with the correlation length ξ in terms of equation (2), the dominant scaling with ξ^3^ can be confirmed for the simpler NPSG structures. The hierarchical HPNG structures do not show a simple scaling, possibly due to considerable variations in several parameters like the second-stage porosity on smaller length scales.

**Figure 8:**
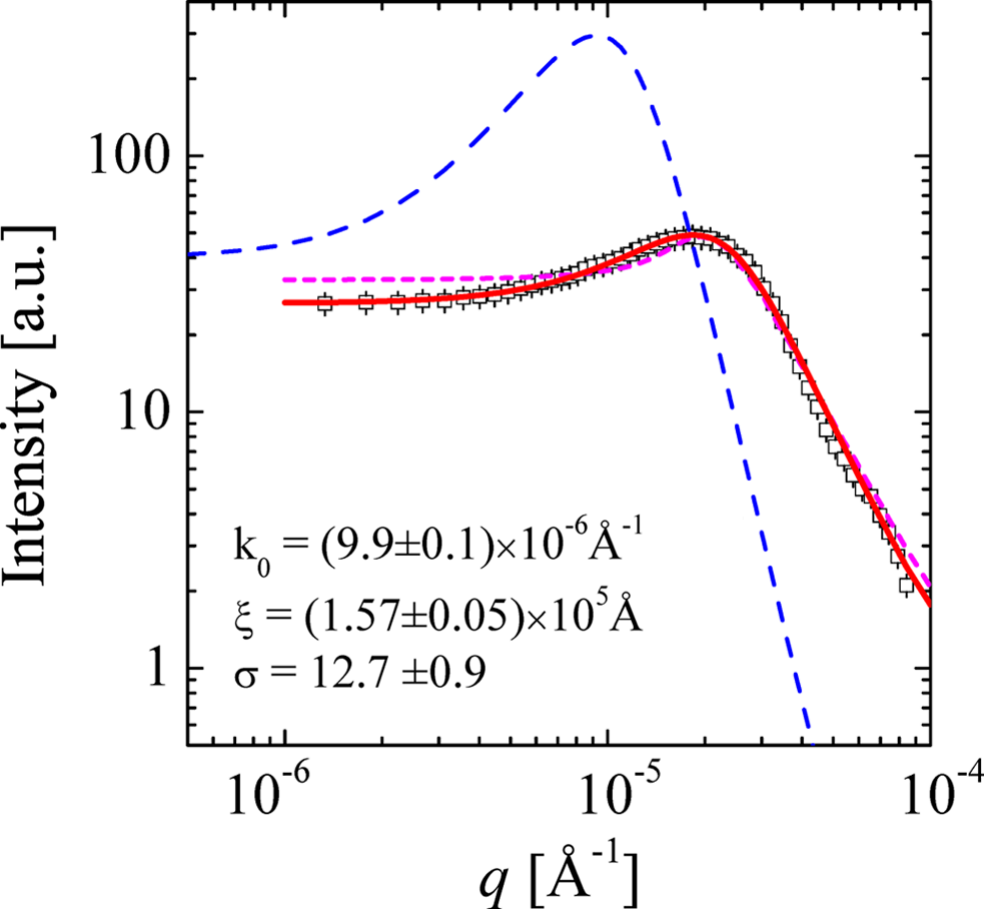
The SALS profile for a porous material [data from Setshedi *et al.* (2020 ▸)] as described by Frielinghaus (2026*a* ▸) (dashed pink line, without stretching). Now, a stretch allows the diffuse scattering to be modelled as well, as depicted by the solid red line. The corresponding bulk scattering is indicated by the dashed blue line (same σ).

The last example is a surface scattering example of a porous material (Setshedi *et al.*, 2020 ▸) that appears for coherent multiple scattering. The simple three-dimensional model has been applied already by Frielinghaus (2026*a* ▸) but the diffuse scattering has not been modelled well. With the current approach [equation (10)], a much better agreement is obtained, as depicted in Fig. 8 ▸. The diffuse scattering at small *q* and the peak shape are described in much better detail. In any case, the high-*q* asymptote is *q*^−2^. From the comparison with the bulk scattering, it becomes evident that the peak position is shifted by a factor of 2. The product *k*_0_ξ lies in a similar range as discussed above.

We have now found several examples where the stretching factor σ lies in the approximate range of 3 to 10. For these cases we also discussed the real-space correlation function (see Fig. 4 ▸). Then, the first neighbouring pore structure is highly correlated to the solid structure, while at larger distances the correlation function decays quickly (due to the small ξ). This scenario has a meaning for the dealloying process (Riedel *et al.*, 2023 ▸) in the two cases of NPSG and HNPG (porous gold), where the silver in the gold alloy is extracted from the solid material through the next neighbouring pore. At that distance the stoichiometry matters greatly, while at large distances the correlations are lost. Similarly, one could argue, for condensation-like and ink-drying (Setshedi *et al.*, 2020 ▸) reactions after printing, that at short distances the stoichiometry matters and so the condensation material becomes highly correlated to the next neighbouring pore (or surrounding solvent *etc.*). This scenario is characterized by the conditions *k*_0_ξ ≲ π and 3 ≲ σ ≲ 10.

The newly introduced stretching parameter σ is purely heutristic and is not simply related to a physical parameter. The case σ = 1 means that the original theory – the extension of the Teubner–Strey theory to arbitrary dimensions – determines the level of the diffuse scattering in terms of the two structural parameters *d* and ξ only. Here, rather strong fluctuations at relatively large distances (compared with the domain spacing *d*) keep the diffuse scattering rather high. The other limit σ = ∞ (in practice, values of 10 and above) describes the compensation of the solid material fluctuations by the first neighbouring pore fluctuations (or refers to two domains with different materials). Here, the stoichiometry in the process of forming the domains did not allow fluctuations over length scales of a few domain spacings *d*. Thus, the limits of σ explain the physical meaning well, while intermediate values are not that strictly related.

## 5. Summary

Porous and/or bicontinuous structures are often characterized using small-angle scattering. In the scattering profile a distinct peak appears that is connected to two structural parameters: the domain spacing *d* (with a preferred wavevector of magnitude *k*_0_ = 2π/*d*) and the correlation length ξ. A high-*q* Porod scattering with a power law *q*^−1−*n*^ is also often observed. In the classical theory, which is a generalization of the well known Teubner–Strey theory for arbitrary dimensions *n*, the diffuse scattering level is highly linked to the structural parameters *k*_0_ and ξ. A scenario for porous and/or bicontinuous structures with a suppressed diffuse scattering has been described and explained. The structural parameters usually describe a broad peak with *k*_0_ξ ≲ π. The new expressions now introduce a stretching factor σ that enhances the separation of the diffuse scattering from the peak scattering. It often lies in the range 3 ≲ σ ≲ 10. The explanation of this scenario is a strong correlation of the solid material to the next neighbouring pore (or domain). The stoichiometry is conserved locally in dealloying, condensation-like and drying processes and thus keeps a stronger correlation. In contrast, a classical microemulsion does not obey a very strict stoichiometry locally and long-range fluctuations are possible. This difference explains the low and high levels of diffuse scattering in the two different scenarios.

A rather heuristic approach for stretching of the correlation peak by the parameter σ is described for bulk and surface scattering. The concept does not physically strictly claim that the duality of the original expressions is maintained by an identical stretching factor σ for bulk and surface scattering. At this point, more experiments are needed.

For dimensionalities close to 3, equations (9) and (10) present a modification of the original theory with arbitrary stretching σ *and* with expressions free from arbitrary hypergeometric functions. This allows the implementation of the simple functions in software like *OriginPro* from OriginLab without the need for mathematical libraries and/or compilation. As I have demonstrated, the variation of the bare hypergeometric peak function with respect to *n* = 3 is small, and indeed negligible compared with the asymptotic function when the exact dimensionality is off by a maximum of, say, 0.5. So, especially for σ = 1 (no stretching), a simpler expression of the original generalized Teubner–Strey theory is found.

## Figures and Tables

**Figure 7:**
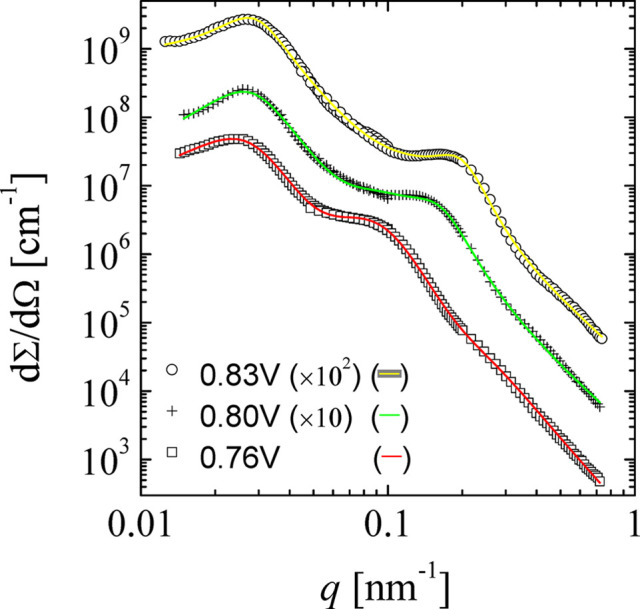
SAXS profiles for hierarchical nanoporous gold (HNPG) [data from Riedel *et al.* (2023 ▸)] from different dealloying potentials *E*_D_. The measurements are indicated by separate symbols as given in the legend. Shift factors are indicated in brackets. The modelling is depicted by continuous lines, coloured as given in the legend.

**Table 1:** Parameters of the scattering curves for the description of experimental data displayed in Figs. 5 ▸–7 ▸ ▸ The parameters relate to equations (9) and (21). *A*_1_ and *A*_3_ have units of intensity and units of *q*^ε^ or *q*^*n*+1^, respectively. The parameters ε, *n* and σ are unitless. The HNPG samples are described by two peaks with the parameters given on two lines. Typical error bars lie in the range of ± 10% or better.

Sample	*A*_1_ (to scale)	ε	*A*_2_ (to scale)	*n*	*k*_0_ (Å^−1^)	ξ (Å)	σ	*A*_3_ (to scale)	*R* (Å)
PG180	2.9 × 10^12^	7	3.6 × 10^29^	3.1	0.009	400	500	1.4 × 10^24^	60
PGF200	4.1 × 10^20^	3.5	6.6 × 10^28^	2.6	0.011	200	4	1.2 × 10^23^	100
NPSG, 500°C	0	–	1.2 × 10^7^	3	0.013	203	2.6	1.2	45
NPSG, 400°C	0	–	3.4 × 10^6^	3	0.028	120	1.0	1.6	29
NPSG, 300°C	0	–	1.5 × 10^5^	3	0.051	62	3.6	4.0	11
HNPG, *E*_D_ = 0.83 V	0	–	8.4 × 10^5^	3	0.028	115	1	0	–
Second peak	0	–	1.1 × 10^2^	3	0.194	19	10	3.2	11
HNPG, *E*_D_ = 0.80 V	0	–	1.4 × 10^5^	3	0.026	92	10	0	–
Second peak	0	–	3.1 × 10^2^	3	0.157	20	10	8.1	14
HNPG, *E*_D_ = 0.76 V	0	–	5.2 × 10^5^	3	0.025	78	10	0	–
Second peak	0	–	1.2 × 10^4^	3	0.085	21	10	11	6.1
